# A Look at the Grouping Effect on Population-level Risk Assessment of Radon-Induced Lung Cancer

**DOI:** 10.5539/gjhs.v5n6p1

**Published:** 2013-07-22

**Authors:** Jing Chen, Deborah Moir

**Affiliations:** 1 Radiation Protection Bureau, Health Canada, Ottawa, Canada

**Keywords:** radon, smoking, lung cancer

## Abstract

On the basis of considerable knowledge gained by studying health effects in uranium and other underground miners who worked in radon-rich environments, radon exposure has been identified as a cause of lung cancer. Recent pooled analyses of residential studies have shown that radon poses a similar risk of causing lung cancer in the general public when exposure occurs at generally lower levels found in homes. With the increasing accessibility of statistical data via the internet, people are performing their own analyses and asking why, in some cases, the lung cancer occurrence at the community level does not correlate to the radon levels. This study uses statistical data available to the general public from official websites and performs simple analyses. The results clearly show the difficulty in linking observed lung cancer incidence rates at the provincial/territorial level, with possible cause, such as smoking or radon exposure. Even the effect of smoking, a well-documented cause of lung cancer, can be overlooked or misinterpreted if the data being investigated is too general (i.e., summary data at population level) or is influenced by other factors. These difficulties with simple comparisons are one of the main reasons that epidemiological studies of lung cancer incidence and radon exposure requires the use of cohorts or case controls at the individual level as opposed to the more easily performed ecological studies at the population level.

## 1. Introduction

Epidemiological studies of uranium and other types of miners have shown a strong relationship between lung cancer mortality and radon exposure (National Research Council, 1988, 1999). These studies were typically of the cohort-type, meaning the population studied was classified according to past exposure history and followed forward in time to observe the rates of various causes of death. These studies compared the rates of lung cancer in miners who worked in radon-rich environments to the general male population. Typically such studies had good accuracy and included data on radon levels, exposure durations and smoking habits of the individuals in the study, which were factored into the analysis.

Historically, the data from the miner studies have been extrapolated to the levels typically found in residential homes and have suggested a risk exists for residents of some houses as well (National Research Council, 1999; UNSCEAR, 2006). Unfortunately direct data on residential risk from radon has been quite limited with most studies comparing lung cancer rates and mean radon exposures in various geographical areas without specific data on individuals. These types of ecological studies suffer from several weaknesses, including biases acting within a population group caused by inadequate control of confounding factors, the assignment of group exposure levels to all individuals of the group, use of crude estimates of population exposure and biases from the mobility of individuals, all of which make conclusions difficult. Ecological studies are often used because they are easy, quick and relatively inexpensive; however, because their results can be difficult to interpret, they are best used as an indicator of the need for a second more carefully designed study if strong associations are indicated.

In the field of epidemiology, the fact is well recognized that ecologic or population-level associations are not necessarily consistent with those measured at the individual-level. Unlike ecological studies, case-control studies link individual outcome events (i.e. lung cancer incidence in this context) to individual exposure (i.e. radon exposure), major affecting factors (such as smoking) and other covariate histories; with all data detailed at individual level. Case-control studies have also been conducted in several countries to try to estimate the risk of lung cancer from residential radon exposure; however, in the past none of these studies was large enough to reliably assess the risks. Such studies are often strongly influenced by the use of data from urban areas where radon concentrations tend to be lower compared to rural areas due to underlying geology and because more people live in apartments where radon levels tend to be reduced. Greater statistical power is needed to correct for these factors and can be accomplished by combining the data from several studies. In 2004 and 2005 researchers in Europe and North American conducted independent pooled case-control studies of lung cancer incidence and radon exposure in residential homes (Darby et al., 2005; Krewski et al., 2006). Both pooled studies indicated an increased risk of lung cancer associated with radon exposure at levels found in some homes.

In 2011 Health Canada completed an extensive two-year survey of residential radon levels in houses across the country. This Cross Canada Survey of Radon Concentrations in Homes provided test results for approximately 14,000 houses and identified areas where a higher percentage of homes were expected to be above Canada’s National Radon Guideline of 200 Bq/m^3^. The data from this survey was subsequently used by Health Canada to reassess the number of lung cancer deaths in Canada due to radon exposure. This revised estimate of 16% points to approximately 3,000 deaths each year from radon with most of these due to the synergistic interaction between radon exposure and smoking (Chen et al., 2012). With such a large number of lung cancers occurring, Health Canada is often asked why regions of the country with high radon levels would not have significantly more lung cancers in comparison to other regions. Sceptics of the health risks associated with radon exposure have asked why the overall lung cancer occurrence by province would not correlate to a corresponding occurrence of radon levels with smoking incidence taken into consideration. While perhaps this might seem to be a reasonable assumption to make, from the previous discussion of epidemiological studies performed in the past, it can be seen to be a gross over-simplification of the analysis needed in order to show a correlation. The dependence of the effects of radon exposure on various factors, such as age at, level and length of exposure, past exposure history, as well as exposure to smoking, implies that an analysis cannot simply be made using mean radon exposures and lung cancer rates for a specific area.

To illustrate the risk of over-simplifying the data, in this publication we have attempted to show how simple comparisons of lung cancer rates with a well documented cause of lung cancer, such as smoking, as well as radon concentration, can lead to a conflicting conclusion of the risk.

## 2. Method

**Figure 1:**
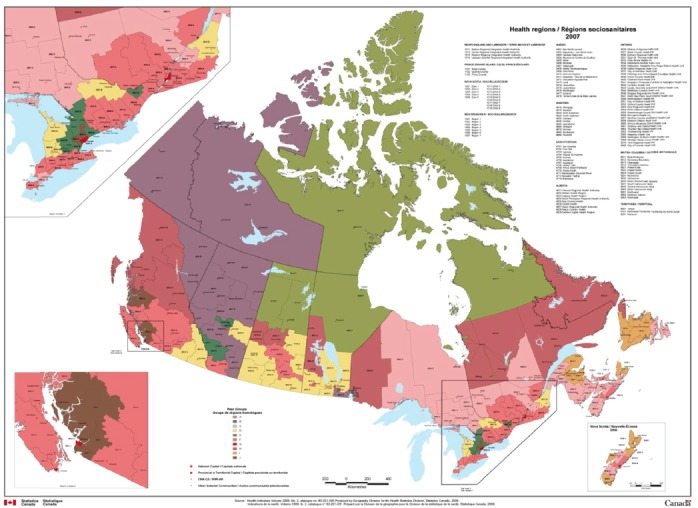
Health regions and peer groups in Canada (for details, visit Statistics Canada Website) (Statistics Canada 2011)

Canada is currently divided into 123 health regions defined by the provincial ministries of health as administrative areas (Statistics Canada, 2011). Statistics Canada has used a statistical method to determine peer groups and assign health regions to peer groups to achieve maximum statistical differentiation between health regions. Health regions were grouped into peer groups in order to effectively compare health regions with similar socio-economic characteristics. Twenty-four variables were chosen to cover as many of the social and economic determinants of health as possible, using data collected at the health region level mostly from the Census of Canada. Variables considered include basic demographics (such as population and ethnicity), living conditions (such as housing information, and income inequality), and working situation (such as the unemployment rate). There are currently ten peer groups identified by letters A through J, as shown in Figure 1.

Following the launch of the National Radon Program in 2007, Health Canada started a national residential radon survey in April 2009 to gain a better understanding of radon concentrations in homes across the country (Health Canada, 2011). In this survey, long-term (3-months or longer) radon measurements were completed in roughly 14,000 homes in 121 health regions (these administrative areas are defined by the provincial ministries of health and subject to change which accounts for the difference in the number of health regions currently studied) across Canada. The proportions of Canadian homes with different ranges of radon concentration used in the current study are based on the Health Canada national residential radon survey roughly uniformly distributed in 121 health regions across Canada (Health Canada, 2011). The survey was conducted over 2 years during the heating seasons (October to April) of 2009/10 and 2010/11. Long-term radon measurements were performed in all the homes surveyed. The observed radon concentrations in Canadian homes follow a log-normal distribution (Chen et al., 2012). Because of the wide distribution in radon concentrations, a central estimate alone, such as arithmetic mean or geometric mean, will not be able to represent the distribution. Therefore, the percentage of homes above 200 Bq/m^3^ was chosen to characterise the radon exposure situation in a given health region. It was assumed that males and females were distributed equally in the various radon concentration ranges.

Canadian cancer incidence data (Table 103-0404) are available on the website of Statistics Canada for the most recent years up to 2007 (Statistics Canada, 2013a). Canadian smoking statistics can be found in Table 105-0501 on the Statistics Canada website (Statistics Canada, 2013b). The most relevant indicator to the present study is the percentage of current daily smokers in a given administrative unit. Even though long term smoking history is known to be more relevant to lung cancer development, the smoking statistics are available to the general public only for recent years, from 2003 to 2011. This study uses only statistical data available to the general public from official websites.

In the statistical analysis conducted here, linear regression is used. The R^2^ coefficient is a statistical measure of how well the regression line approximates the real data points. R^2^ = 1 indicates that the regression line perfectly fits the data while R^2^ = 0 indicates no linear relationship, i.e. we cannot predict one variable from the other.

## 3. Results and Discussion

**Figure 2:**
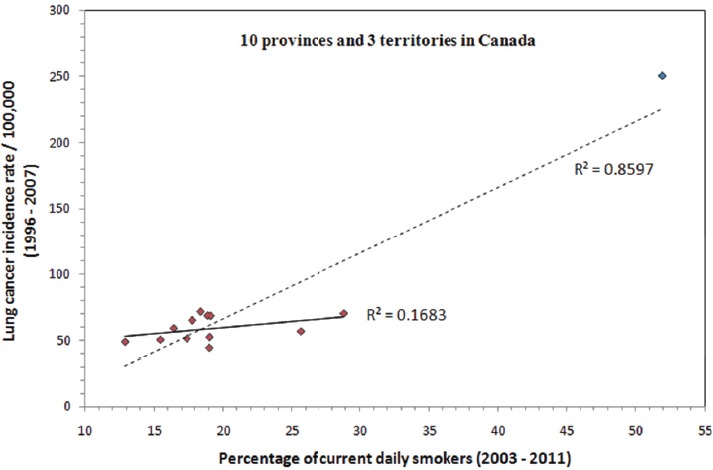
Statistics of lung cancer incidence as a function of the percentage of current daily smokers for the 10 provinces and 3 territories in Canada

Summary statistics of lung cancer incidence, percentage of current daily smokers among a population and percentage of homes above 200 Bq/m^3^ are given in Table 1 for the 10 provinces and 3 territories in Canada, respectively. Even though an enormous body of scientific evidence clearly documents that cigarette smoking is the major cause of lung cancer (IARC 2004), provinces/territories, such as Quebec, Prince Edward Island, Nova Scotia and New Brunswick, with higher lung cancer incidence rates do not necessarily correlate with higher smoking rates as shown in Table 1, making it clear that many factors must contribute to the development of lung cancer. As can be seen, this type of simple tabular comparison can be deceiving, and thus a graphical view of lung cancer incidence in relation to the percentage of current daily smokers is presented in Figure 2. Clearly, there is a strong relationship between lung cancer incidence rate and the smoking rate with R^2^ = 0.86. However, the strong relationship with smoking is dominated by the data from Nunavut. If the data from Nunavut is excluded as an outlier, a correlation (R^2^ = 0.17) can still be seen between lung cancer and smoking, albeit significantly weaker.

**Table 1:** Summary statistics of lung cancer incidence, percentage of current daily smokers and percentage of homes above 200 Bq/m^3^ for the 10 provinces and 3 territories

	Lung Cancer Incidence per 100,000 (1996 – 2007)	Current daily smoker % (2003 – 2011)	Homes > 200 Bq/m^3^ %
Canada	58.1	16.4	6.9
Newfoundland and Labrador (NL)	44.3	19.0	5.1
Prince Edward Island (PEI)	65.2	17.8	3.5
Nova Scotia (NS)	68.8	18.9	10.7
New Brunswick (NB)	68.6	19.1	19.4
Quebec (QC)	71.8	18.4	8.2
Ontario (ON)	50.5	15.4	4.6
Manitoba (MB)	59.1	16.4	19.4
Saskatchewan (SK)	52.5	19.0	15.7
Alberta (AB)	51.4	17.4	5.7
British Columbia (BC)	48.9	12.9	3.9
Yukon (YT)	56.8	25.6	19.6
Northwest Territories (NT)	70.4	28.8	5.4
Nunavut (NU)	250.5	51.9	0.0

**Figure 3:**
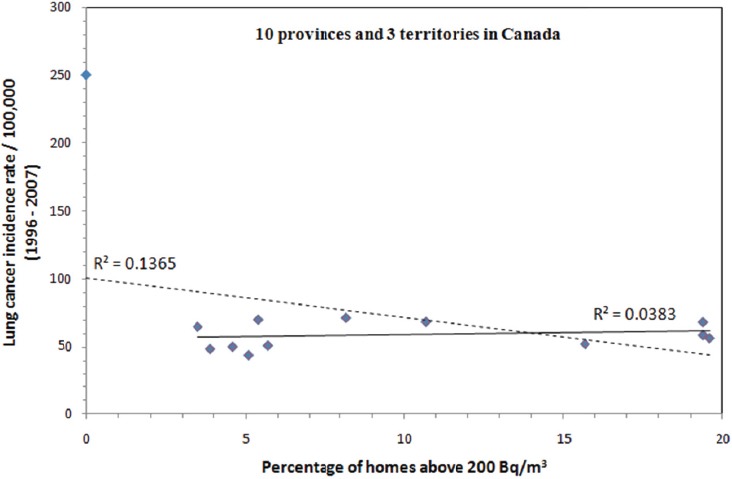
Statistics of lung cancer incidence as a function of the percentage of homes above 200 Bq/m^3^ for the 10 provinces and 3 territories in Canada

Compared to the effect of smoking, we know exposure to radon is a minor cause of lung cancer (the current estimate is 16% of all lung cancers are attributable to radon). As shown in Figure 3, a province/territory of higher lung cancer incidence does not necessarily correlate with more homes above 200 Bq/m^3^. A clear negative association (R^2^ = 0.14) is observed between cancer incidence and radon exposure. This negative association is dominated by the data from Nunavut. However, Nunavut is a unique territory in that many homes are built on stilts because of the permafrost. This architectural factor means many homes in Nunavut will not suffer from infiltration of any radon that is able to find its way to the surface of the earth. For this reason data from Nunavut is not necessarily comparable to the rest of Canada. If we treat the data from Nunavut as an outlier, a very weak but positive relationship (R^2^ = 0.04) can be seen between lung cancer and radon exposure.

**Figure 4:**
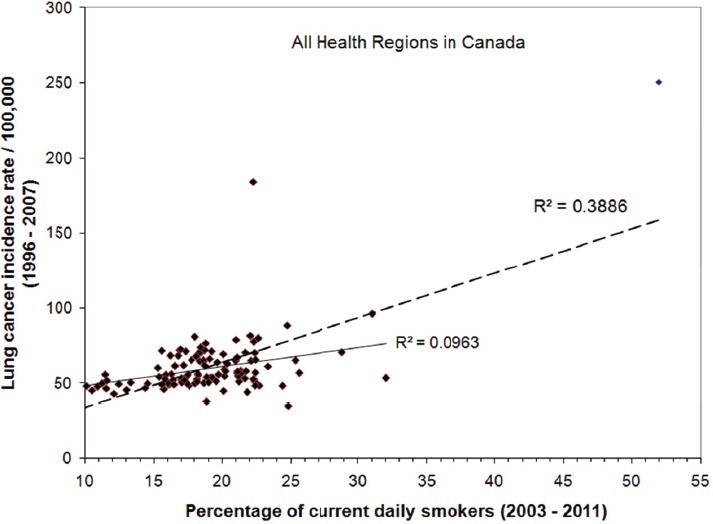
Statistics of lung cancer incidence as a function of the percentage of current daily smokers for all health regions in Canada

**Figure 5:**
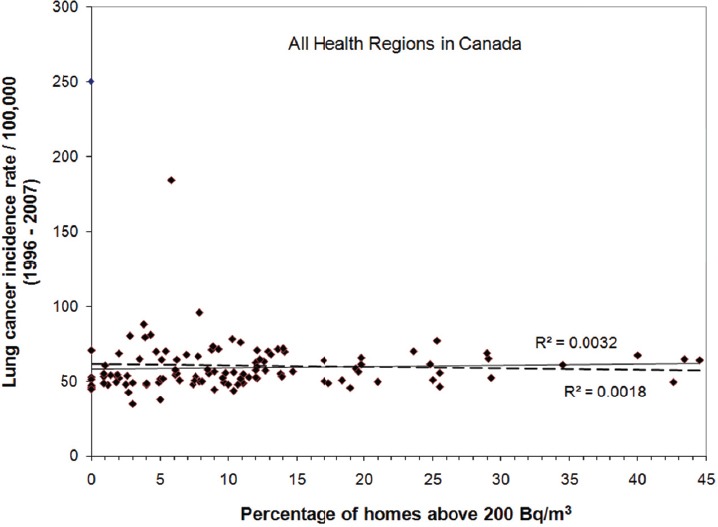
Statistics of lung cancer incidence as a function of the percentage of homes above 200 Bq/m^3^ for all health regions in Canada

While data is most commonly grouped according to provinces and territories, as in the case of the statistics shown in Table 1 above, an alternative grouping that can be used is according to health region. The same dataset presented in Table 1 can be further broken down with the lung cancer incidence shown as a function of percentage of current daily smokers for all health regions in Canada, as illustrated in Figure 4. Although it is clear from this graph that some health regions with higher smoking rates may not have a higher rate of lung cancer incidence, there is still, statistically speaking, a clear positive association (R^2^ = 0.39) between lung cancer incidence and smoking rate. Again, if the data from Nunavut is treated as an outlier, a small correlation (R^2^ = 0.10) can still be seen between lung cancer and smoking. At the health region level, the smoking effect can still be seen; however, the observed association between lung cancer and smoking becomes significantly weaker than that at the provincial level. For the grouping at the health region level, the statistics do not show any association (R^2^ = 0.00) between lung cancer and radon exposure, either with or without data from Nunavut, as shown in Figure 5. This is hardly surprising, given the weak association seen for smoking rate and lung cancer as the size of the data pool becomes smaller at this level of detail. Similar observations/examples were given by Greenland and Robins (1994) in their commentary on biases, misconceptions and counterexamples in ecologic studies. In that study they showed a positive correlation existed for the linear regression of regional lung-cancer rates and regional mean cigarette consumptions and a negative exposure-response relationship between the regional lung-cancer rates and regional radon concentrations. As they pointed out, there are many potential confounders and the associated bias evaluation can be especially difficult in ecologic studies of geographic regions because of many potentially interacting covariates that may differ across regions. It is very difficult to rule out those biases with summary data at the population level.

**Figure 6:**
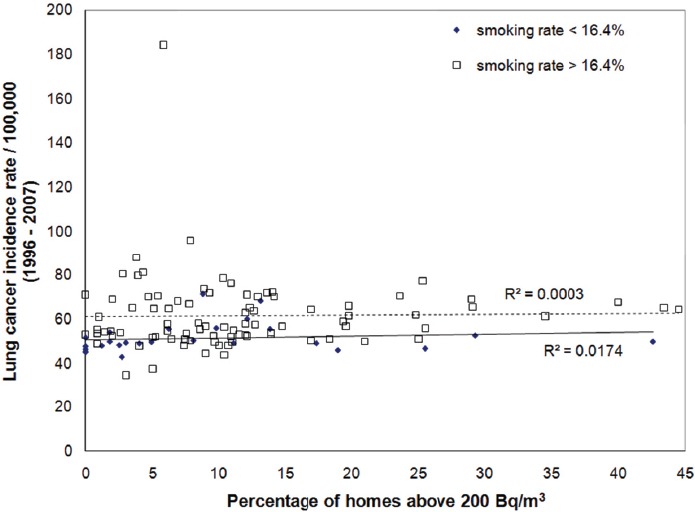
Statistics of lung cancer incidence as a function of the percentage of homes above 200 Bq/m^3^ for two subgroups: health regions with smoking rates less than 16.4% and health regions with smoking rates above 16.4%

The more than one hundred health regions in Canada can be further grouped into two groups to somewhat reduce the impact of smoking on the results, one with health regions having a smoking rate less than the national average of 16.4% and another with health regions having a smoking rate higher than the national average. As shown in Figure 6, while health regions with higher smoking rates could not demonstrate any association (R^2^ = 0.00) between lung cancer and radon exposure, a very weak but positive relationship (R^2^ = 0.02) can be seen between lung cancer and radon exposure among health regions with smoking rates less than 16.4%.

**Figure 7:**
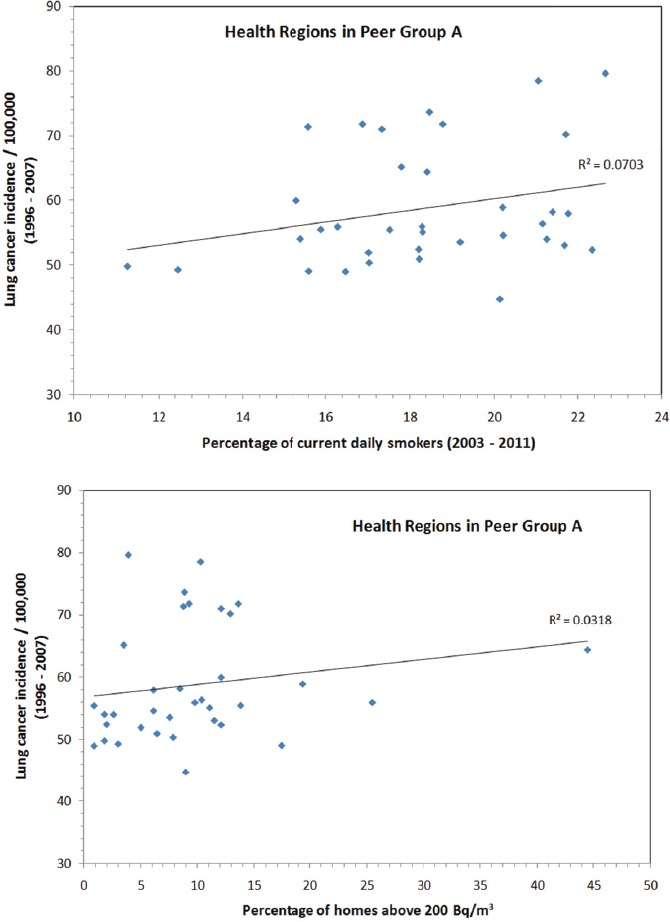
Statistics for health regions in Peer Group A, upper panel - lung cancer incidence as a function of the percentage of current daily smokers; lower panel - lung cancer incidence as a function of the percentage of homes above 200 Bq/m^3^

Because many socio-economic characteristics affect cancer incidence, it is worthwhile to analyse data from a peer group of health regions having similar socio-economic characteristics. Among the 10 peer groups in Canada as defined by Statistics Canada, Peer Group A contains the most health regions across Canada. With a total of 35 health regions in Peer Group A, it is then possible to investigate the effect of various subgroupings. Data for Peer Group A are presented in Figure 7 as a function of the percent of current daily smokers and the percentage of homes with radon levels above 200 Bq/m^3^. Generally speaking, weak associations can be observed for both smoking and radon exposure with lung cancer, with the effect of smoking effect (R^2^ = 0.07) slightly stronger than the effect of radon exposure (R^2^ = 0.03).

**Figure 8:**
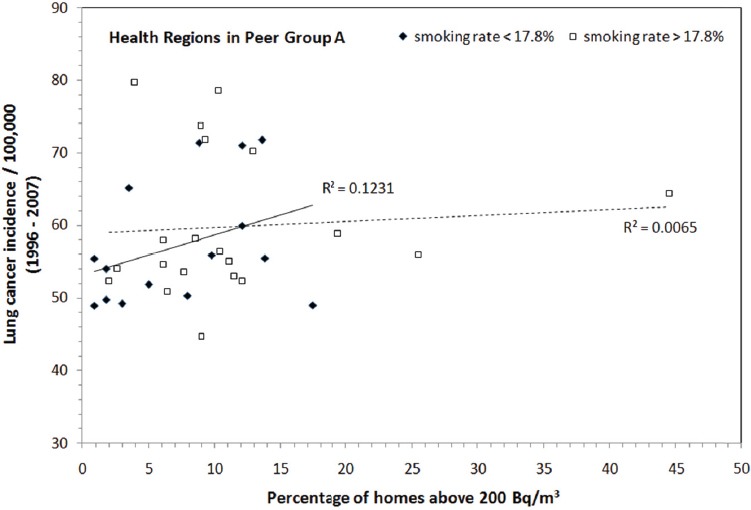
Statistics of lung cancer incidence as a function of the percentage of homes above 200 Bq/m^3^ for two subgroups in Peer Group A: health regions with smoking rates less than 17.8% and health regions with smoking rates above 17.8%

The average percentage of current daily smokers in Peer Group A is 17.8%. If we regroup health regions in Peer Group A into two subgroups based on whether the smoking rate is below or above the average of 17.8%, there are 15 health regions with smoking rates less than the Peer Group’s average. As shown in Figure 8, the association between lung cancer incidence and radon exposure in this subgroup, with relatively lower smoking rates, improved significantly from R^2^ = 0.03 for the entire Peer Group A to R^2^ = 0.12 for the subgroup. This shows that a rather clear association between lung cancer and radon exposure can be observed when smoking is less of a factor in the regions being studied.

**Figure 9:**
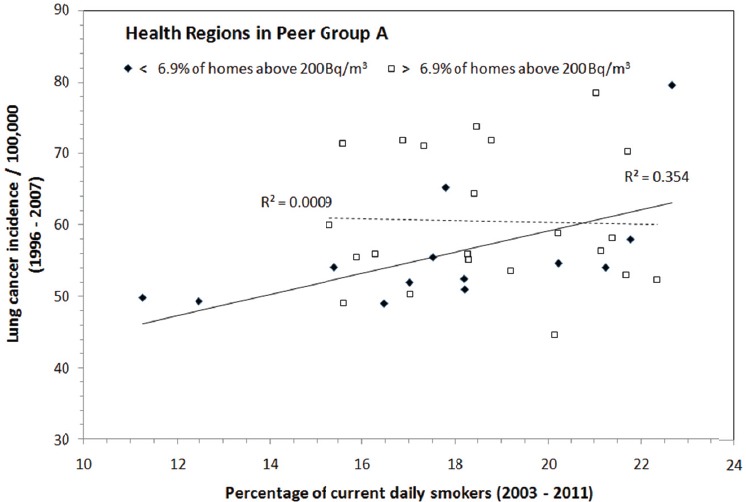
Statistics of lung cancer incidence as a function of the percentage of current daily smokers for health regions with percentages of homes having radon > 200 Bq/m^3^ below or above the national average of 6.9%, respectively

In order to reduce the influence of radon exposure on the results, health regions in Peer Group A are divided into two subgroups based on whether the percentage of homes above 200 Bq/m^3^ in that health region is below or above the national average of 6.9%. As shown in Figure 9, radon exposure is below the national average in 13 health regions of Peer Group A. The association between lung cancer incidence and smoking rate in those health regions increased significantly from R^2^ = 0.07 for the entire Peer Group A to R^2^ = 0.35 for the subgroup of less homes above 200 Bq/m^3^. It is clear that when radon exposure is less of an influence, a stronger association for smoking-induced lung cancer can also be demonstrated.

## 4. Conclusions

While primarily interested in the effect of radon exposure at the individual level, the information most often available for analysis is population-level summary data, which if used without understanding of the limitations can produce inaccurate conclusions. The most significant shortcoming of ecological studies is the fact that regional average exposure levels are assigned to individuals of a population group, but the average risk determined for the population does not correlate well with the average exposure. Compounding this issue is that the magnitude and direction of the ecological bias could vary depending on the population level selected or how individuals are grouped. These biases often cannot be eliminated by the addition of more data at the population–level, therefore population-level analyses or ecological studies should be primarily reserved for hypothesis generation. Results from the current study clearly show the difficulty in linking observed lung cancer incidence rates at the provincial/territorial level, with cause, such as smoking or radon exposure. Even the influence of smoking, a well-documented cause of lung cancer, can be overlooked or misinterpreted if the data being investigated is too general or too crude (i.e., using summary data at provincial or regional level) or is influenced by other factors which cannot be effectively controlled with data summarised at the population level. These difficulties with simple population level comparisons are one of the main reasons that epidemiological studies of lung cancer incidence and radon exposure require the use of cohorts or case controls at the individual level as opposed to the more easily performed ecological studies at the population level. Although also subject to some biases, it is individual-level analyses, i.e. case-control epidemiologic studies, that should be used to test a hypothesis. In the case of environmental radon exposure, involving low doses and therefore subject to large statistical uncertainty, even a case-control study at the individual-level suffers from problems and requires a very large group of individuals in order to be able to observe a statistically significant number of effects.
